# Evaluation of the TEG^® ^platelet mapping™ assay in blood donors

**DOI:** 10.1186/1477-9560-5-3

**Published:** 2007-02-20

**Authors:** Louise Bochsen, Bo Wiinberg, Mads Kjelgaard-Hansen, Daniel A Steinbrüchel, Pär I Johansson

**Affiliations:** 1 Department of Clinical Immunology, Rigshospitalet, University of Copenhagen, Copenhagen, DK-2100, Denmark; 2 Department of Small Animal Clinical Sciences, The Small Animal Hospital, Faculty of Life Sciences, University of Copenhagen, Frederiksberg, DK-1870, Denmark; 3 Department of Cardiothoracic Surgery, Rigshospitalet, University of Copenhagen, Copenhagen, DK-2100, Denmark

## Abstract

**Background:**

Monitoring of antiplatelet therapy in patients at cardiovascular risk is difficult because existing platelet function tests are too sophisticated for clinical routine. The whole blood TEG^® ^Platelet Mapping™ assay measures clot strength as maximal amplitude (MA) and enables for quantification of platelet function, including the contribution of the adenosine diphosphate (ADP) and thromboxane A2 (TxA2) receptors to clot formation.

**Methods:**

In 43 healthy blood donors, the analytical (CV_*a*_) and inter-individual variability (CV_*g*_) of the TEG^® ^Platelet Mapping™ assay were determined together with platelet receptor inhibition in response to arachidonic acid (AA) and ADP.

**Results:**

The CV_*a *_of the assay for maximal platelet contribution to clot strength (MA_*Thrombin*_) was 3.5%, for the fibrin contribution to clot strength (MA_*Fibrin*_) 5.2%, for MA_*AA *_4.5% and for MA_*ADP *_it was 6.6%. The MA_*Thrombin *_CV_*g *_was 2.8%, MA_*Fibrin *_4.7%, MA_*AA *_6.6% and for MA_*ADP *_it was 26.2%. Females had a higher MA_*Thrombin *_compared to males (62.8 vs. 58.4 mm, p = 0.005). The platelet TxA2 receptor inhibition was 1.2% (range 0–10%) and lower than for the ADP receptor (18.6% (0–58%); p < 0.0001).

**Conclusion:**

The high variability in ADP receptor inhibition may explain both the differences in response to ADP receptor inhibitor therapy and why major bleeding sometimes develops during surgery in patients not treated with ADP receptor inhibitors. An analytical variation of ~5 % for the TEG^® ^enables, however, for routine monitoring of the variability in ADP receptor inhibition and of antiplatelet therapy.

## Background

Antiplatelet therapy is important for cardiovascular medicine. The efficacy of aspirin in both primary and secondary prevention of myocardial infarction and stroke and hence cardiovascular death is established [1,2] and the addition of a platelet adenosine diphosphate (ADP) receptor inhibitor, clopidogrel, further reduces these risks [3,4]. Yet, platelet function tests (PFT) demonstrate that subgroups of patients fail to develop the anticipated antiplatelet effect of aspirin and/or clopidogrel [5,6]. The PFT are, however, not applicable for routine purposes and hence for clinical monitoring of antiplatelet therapy [7].

The whole blood Thrombelastograph (TEG^®^) Platelet Mapping™ assay measures clot strength, maximal amplitude (MA), reflecting maximal platelet function, and detects the reduction in platelet function, presented as percentage inhibition, by both aspirin [8] and clopidogrel. This study evaluated the analytical (CV_*a*_) and inter-individual (CV_*g*_) variation of the assay and the platelet aggregation response, expressed as inhibition, to arachidonic acid (AA), representing a measure of the cyclooxygenase-1 (COX-1) activity, and to ADP in healthy blood donors.

## Methods

The study was in accordance with the Helsinki 2 Declaration and the participants provided informed consent prior to any study related activity. Forty-three Danish blood donors, not taking any medication 10 days prior to the investigation were included in the study. Blood was drawn in citrate (9 volumes of blood into 1 volume of 0.129 M citrate, Vacutainer system, BD Biosciences, Plymoth, UK) and into heparin (17 IU/ml blood, Vacutainer system, BD Biosciences, Plymoth, UK).

### Thrombelastography

The TEG^® ^Platelet Mapping™ assay (Haemoscope Corporation, Niles, Illinois, US) relies on evaluation of clot strength to enable a quantitative analysis of platelet function. The maximal haemostatic activity is measured by a kaolin activated whole blood sample treated with citrate. The following measurements are performed with heparin to eliminate thrombin activity: Reptilase and Factor XIII (Activator F) generate a cross-linked fibrin clot to isolate the fibrin contribution to the clot strength [9]. The contribution of the ADP or ThromboxaneA2 (TxA2) receptors to the clot formation is provided by the addition of ADP or AA.

Blood was analyzed according to instructions (Haemoscope Corporation. TEG Guide to Platelet Mapping. Monitor anti-platelet therapy, 2004). Both analyzer (series 5000) and the reagents were from Haemoscope Corporation.

**Figure 1:**
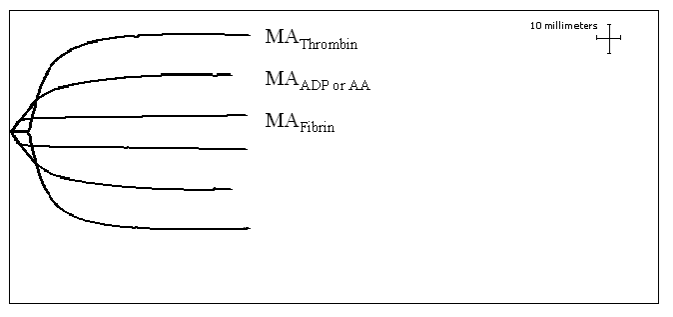
Profiles of the TEG^® ^Platelet Mapping™ assay parameters MA_*Thrombin*_, MA_*ADP*/*AA*_, and MA_*Fibrin*_.

For maximal clot strength (MA_*Thrombin*_) one milliliter of citrate-stabilized blood was transferred to a vial containing kaolin and mixed by inversion. Kaolin activated blood (340 μl) was added to a TEG^® ^cup containing 20 μl of 0.2 M CaCl_2_. To generate a whole-blood fibrin cross-linked clot, representing only the fibrin contribution included in the clot strength measurement, heparinized blood (360 μl) was transferred to a TEG^® ^cup containing 10 μl Activator F; MA_*Fibrin *_(Fig. 1). The contribution of the P2Y12 receptor, or the COX-1 pathway, to the clot formation is assessed by the addition of ADP or AA. Therefore, AA and ADP, respectively, are added to Activator F to measure the degree of ADP receptor and thromboxane A2 induced platelet aggregation. Heparinized blood (360 μl) was added to a TEG^® ^cup in the presence of the Activator F and agonist, 10 μl ADP (2 μM, final concentration) yielding MA_*ADP *_or 10 μl AA (1 mM, final concentration) for the MA_*AA*_. The platelet inhibition in response to the agonist is calculated from platelet aggregation: [(MA_*ADP *_- MA_*Fibrin*_)/(MA_*Thrombin *_- MA_*Fibrin*_) × 100] and % inhibition = (100% - % aggregation).

### Statistical analysis

The coefficient of variation for CV_*a *_and CV_*g *_was calculated for MA_*Thrombin*_, MA_*Fibrin*, _MA_*AA *_and MA_*ADP*_[10]. Data are presented as mean ± SD or as mean with range. Comparisons were made by Wilcoxon rank sum tests and a p-value < 0.05 was considered statistically significant.

## Results

The MA_*Thrombin *_was 60.9 ± 5.3 mm (Table 1). When comparing genders, females had both the higher MA_*Thrombin *_and MA_*Fibrin *_(62.8 vs.58.3 mm, p = 0.005 and 8.1 vs. 6.6 mm; p = 0.035, respectively). The platelet aggregation in response to AA, expressed as the percentage of natural inhibition of the TxA2 receptor, varied between 0 and 10% with an average of 1.2%. The platelet aggregation in response to ADP, expressed as the percentage of natural inhibition of the ADP receptor, varied between 0 and 58% with an average of 18.6% being higher than for the TxA2 receptor, p < 0.0001. No significant difference was, however, found between genders with regard to inhibition of the TxA2 (1.3 vs. 1.1%, p = 0.71) or ADP receptors (18.2 vs. 19.4%, p = 0.41).

**Table 1:** TEG^® ^Platelet Mapping™ assay variables (see text) and percent platelet receptor inhibition for healthy blood donors.

	**All donors**	**Females **(n = 27)	**Males **(n = 16)
MA_*Thrombin*_	60.9 (4.5)	62.8 (5.3)	58.4 (5.4) *
MA_*Fibrin*_	7.5 (2.7)	8.1 (2.8)	6.6 (2.2) *
MA_*ADP*_	51.1 (8.1)	52.7 (7.8)	48.5 (8.1)
MA_*AA*_	64.6 (4.7)	66.1 (4.5)	62.5 (4.3)
% ADP RI^a^	18.6 (0–58.1)	18.2 (0–58.1)	19.4 (0–37.5)
% TxA2 RI^a^	1.2 (0–10.1)	1.3 (0–9.8)	1.1 (0–10.1)

Values are mean and SD or range

^a^RI = receptor inhibition

*Comparison between female and male donors, p < 0.05

The CV_*a *_values were 3.5% for MA_*Thrombin*_, for MA_*Fibrin *_it was 5.2%, for MA_*ADP *_6.6 %, and for MA_*AA *_4.5%. The CV_*g *_for MA_*Thrombin *_was 2.8%, for MA_*Fibrin *_it was 4.7% and for MA_*AA *_6.6%, whereas the CV_*g *_for the MA_*ADP *_was 26.2% (Table 2).

**Table 2:** Analytical (CV_*a*_) and inter-individual (CV_*g*_) variation of the TEG^® ^Platelet Mapping™ assay variables (see text) in blood donors.

	MA_*Thrombin*_	MA_*Fibrin*_	MA_*ADP*_	MA_*AA*_
CV_*a *_(%)	3.5	5.2	6.6	4.5
CV_*g *_(%)	2.8	4.7	26.2	6.6

## Discussion

The analytical variation (CV_*a*_) of the TEG^® ^Platelet Mapping™ assay was ~5% in alignment with the findings of Craft et al. [9] investigating 120 subjects and concluding that this point of care assay makes it possible to conduct large scale comparative studies on the degree of platelet inhibition and patient outcome. Conventional aggregometry, representing the current "gold standard" for measuring platelet reactivity to ADP and AA and to assess the effect of antiplatelet agents, is valuable for the experienced and specialised laboratory.

The low analytical variation of the TEG^® ^Platelet Mapping™ assay found in this study may reflect the use of whole blood, obviating pre-analytical and analytic factors such as platelet count and size, preparation of platelet rich plasma (PRP), including centrifugation steps [11,12]. The platelet function analyzer 100 (PFA-100), likely the most widely used point of care test for platelet inhibition, measures the time required for blood under simulated high shear flow to occlude a collagen/epinephrine or a collagen/ADP-coated aperture inserted in a plastic membrane. The PFA-100 is valuable for evaluation of platelet inhibition due to AA, while platelet inhibition due to ADP remains unsolved [13,14]. Duplicate testing, however, is a requirement to obtain reliable results [14].

The thrombin induced clot formation, MA_*Thrombin*_, represents the maximum uninhibited platelet function and was within the normal reference values of 51 to 69 mm, as described by the manufacturer, except for one donor having a MA_*Thrombin *_of 70.1 mm. A gender difference, with females having the higher MA_*Thrombin*_, suggests that females are better protected against bleeding than males [15].

The TEG^® ^Platelet Mapping™ assay enables for evaluation of the respective contribution of the ADP and the TxA2 receptors to clot formation by the addition of the appropriate agonists.

The response in platelet aggregation due to the agonist AA presented as the percentage inhibition of the platelet TxA2 receptor was less than 2% with no difference between gender and lower than the reported value of 14% [8]. However, Gurbel et al. [8] investigated only 6 donors, whereas 43 donors were evaluated in the present study. Genetic polymorphism in the platelet receptors has been reported and we cannot exclude that other differences exist between the two populations.

The variation in platelet aggregation due to ADP stimulation, evaluated as the percentage ADP receptor inhibition, was almost 60% and independent of gender. The inter-individual difference could be attributed to differences in the ADP receptors and to the number of receptors the individual possesses, varying the levels of ADP release, or platelet activation via alternative pathways [6]. Differences in response to ADP receptor inhibitors between individuals have been demonstrated [16,17] and may, at least in part, be due to the difference in platelet reactivity.

Patients treated with the ADP receptor inhibitor clopidogrel are at risk of major bleeding during surgery [18]. Due to the high variation between the donors in ADP receptor inhibition, those with a high natural inhibition may be at risk of developing excessive bleeding during surgery.

The TEG^® ^Platelet Mapping™ assay enables relating the percent platelet inhibition to the individual's maximum uninhibited platelet function. Hereby the individual response to antiplatelet therapy is related to their own maximum uninhibited platelet function of potential therapeutic consequence. A MA_*Thrombin *_value above the normal reference range is associated with an increased risk of thrombotic complications and ischemic events [19,20] implying that individual antiplatelet therapy may reduce the risk of recurrent events and also prevent the risk of bleeding.

Only Caucasian blood donors were studied and the utility of the assay for patients at cardiovascular risk remains to be evaluated. Natural platelet receptor inhibition was investigated and, therefore, the validity of the assay for monitoring patients in antiplatelet therapy was not assessed.

## Conclusion

An analytical variation of ~5 % for the TEG^® ^Platelet Mapping™ assay enables for routine monitoring of antiplatelet therapy in patients at cardiovascular risk. The high natural ADP receptor variability may, in part, explain differences in response to clopidogrel therapy. Furthermore, the high variability in natural ADP receptor inhibition may explain why unexpected bleeding can develop during surgery in some patients as although they are not treated with ADP receptor inhibitors.

## Competing interests

The author(s) declare that they have no competing interests.

## Authors' contributions

LB did all laboratory work, data collection and calculation and contributed in the study design and manuscript preparation. BW, MKH, DAS and PIJ were involved in the statistical analyses and contributed all to the preparation of the manuscript. Additionally, PIJ participated in the design of the study. All authors read and approved the final manuscript.
